# Atypical Kawasaki Disease Complicated With Coronary Artery Aneurysm: A Case Report and Review of Literature

**DOI:** 10.7759/cureus.13589

**Published:** 2021-02-27

**Authors:** Sanjay K Tanti, Sudhir Mishra

**Affiliations:** 1 Department of Pediatrics, Tata Main Hospital, Jamshedpur, IND

**Keywords:** kawasaki disease (kd), incomplete kawasaki disease, coronary artery aneurysm, vasculitis, parotitis, torticollis, intravenous immunoglobulins (ivig)

## Abstract

Background: Kawasaki disease (KD) is an acute, febrile systemic vasculitis of early childhood. A small group of KD patients does not meet the classical presentation of KD, termed incomplete KD. Incomplete or atypical KD patients are usually infants and older children. Because of atypical manifestations of KD, timely diagnosis of KD is difficult, which leads to coronary artery complication

Case presentation: We report the case of a nine-year-old boy who developed fever and right side parotitis with painful cervical lymphadenopathy leading to torticollis as the first symptom of Kawasaki disease (KD). A series of investigations revealed elevated inflammatory markers and aneurysmal dilation of coronary artery on echocardiogram, and thus he was diagnosed with atypical KD. Intravenous immunoglobulin was given and the child responded well. Coronary artery aneurysm resolved by six months.

Conclusion: A high index of suspicion should be maintained in children presenting with fever and unusual manifestations like lymphadenopathy and parotitis, especially where empiric antibiotics were ineffective. Evaluation of cardiac function and coronary artery status with echocardiography is helpful in defining the diagnosis of KD in such cases. As it is a noninvasive test, it should be undertaken at the first possible clinical suspicion.

## Introduction

Kawasaki disease (KD) is an acute febrile systemic vasculitis. It involves mainly medium-sized vessels in infant and younger children [1]. Kawasaki disease is seen in all ethnic groups and countries, with the highest incidence in Asian children [2]. It is the second most common vasculitis of childhood and is the most common cause of acquired heart disease in children in developed countries [3]. In most cases it is self-limited but about 25% of cases experience coronary artery abnormalities if untreated. The etiology of Kawasaki disease is still unknown and no single pathognomonic clinical or laboratory finding for the diagnosis has been identified. KD is a clinical diagnosis based on the sequence in which symptoms and signs appear. Individually the symptoms and signs are not diagnostic. “Classic KD” is defined by the patient presenting with more than five days of fever and showing signs of more than four of the five principal clinical features: polymorphous rash, mucosal changes (including dry, cracked lips and strawberry tongue), extremity changes (including palmar and/or plantar erythema, swelling, and desquamation), bilateral non-purulent conjunctivitis, and cervical lymphadenopathy (≥1.5 cm diameter), usually unilateral [3]. Atypical KD refers to patients who do not meet full criteria and in whom atypical features may be present [3]. They have compatible laboratory findings and no other explanation for their illness. Patients have fever (even less than five days) with only two or three of the cardinal clinical manifestations [4]. It affects children beyond the typical age group, i.e. below six months or more than five years. According to studies from Japan and Canada, KD among older children above nine years old is rare and affects 1% and 9% of patients respectively [5,6]. The diagnosis is more difficult and delayed which leads to coronary artery complication. KD is treated with aspirin and intravenous immune globulin (IVIG) which reduces the incidence of coronary artery involvement from approximately 25% to less than 4% [4].

Here we report an atypical KD, a nine-year-old boy who presented with fever, right side parotitis, and painful cervical lymphadenopathy leading to torticollis complicated with coronary artery aneurysm.

## Case presentation

A nine-year-old boy, previously healthy, was admitted with complaints of cough and cold for seven days, fever for two days, and pain and swelling over the right side of his neck with torticollis for two days. There was no history of head/neck trauma. There was no history of Koch’s contact. The child was immunized for his age as per the Indian Academy of Pediatrics schedule. On examination the child was sick-looking and febrile (temp: 103.3°F); his head was tilted to the right with chin rotation to the left. Pulse rate was 130/min, respiratory rate was 24/min, and blood pressure in right upper limb was 123/67 mmHg. Mild pallor was present. Throat was erythematous and congested. On neck examination his chin was turned towards the left side and a 3x4 cm firm, tender movable mass in the right submandibular region was noted. The right parotid gland was also tender and enlarged. Cardiovascular exam revealed normal peripheral pulses, a quiet precordium with normal heart sounds, and no murmur. There were no bruits heard on auscultation of major vessel regions. Respiratory system was normal. The abdomen was soft on palpation with no distension and tenderness. There was hepatomegaly 3 cm from the right costal margin. There were no rashes or desquamation of the skin. Neurological exam was normal. On the basis of congested throat, fever, submandibular mass and parotid swelling a diagnosis of acute bacterial pharyngitis with cervical lymphadenopathy was made. Differential diagnosis of mumps, acute viral pharyngitis, and abscess in submandibular lymph node was considered and the relevant investigations were sent. As the child was sick-looking, IV antibiotic (ceftriaxone) was started. Initial investigation showed hemoglobin 11.1 gm%, total leukocyte counts (TLC) 13800/mm³ with neutrophilic predominance (P 84%), platelet count 308000/mm³, erythrocyte sedimentation rate (ESR) 32 in the first hour, C-reactive protein (CRP) 18.31 mgm/L, serum amylase 110 IU/L(↑), lipase 18.8 IU/L(N), malarial parasite and dengue serology were negative, liver function and kidney function tests were normal, routine urine examination was normal. Blood and throat swab cultures showed no growth. Virology workup could not be done. High-resolution ultrasound (HRUS) neck showed multiple enlarged right cervical lymph nodes, the largest being 3x4cm² in submandibular regions and there was no evidence of abscess. The right parotid gland was mildly enlarged. A few enlarged lymph nodes were also seen on the left side of the neck.

Fever was persisting even after 96 hours (day six of illness) of antibiotic, and the child developed gastrointestinal (GIT) symptoms of pain abdomen and blood mixed loose stool four to five episodes, so antibiotic were revised in view of no response to initial antibiotic and the child was sick and was put on IV meropenem and amikacin. Repeat blood investigation showed that hemoglobin (10.2 gm%) was decreased. Total leucocyte count (18100/mm³), platelet count (443000/mm³), and CRP (20.6 mg/L) were increased compared to previous reports. Ultrasound of the abdomen was done in view of the patient’s complaints of abdominal pain and to rule out hydrops of the gall bladder; the result was unremarkable.

On day six of admission (day eight of illness) fever was persisting and the child developed bilateral hand and feet edema, congestion of pharynx increased, and his tongue became red with strawberry appearance. At this stage, repeat blood investigation showed increasing platelet count (570000/mm³), CRP concentration 29 mg/L, and ESR 65 mm/1 hour. Hemoglobin (HGB) fell to 9.9 g/dL. White blood cell (WBC) and differential counts were more or less the same.

**Figure 1:**
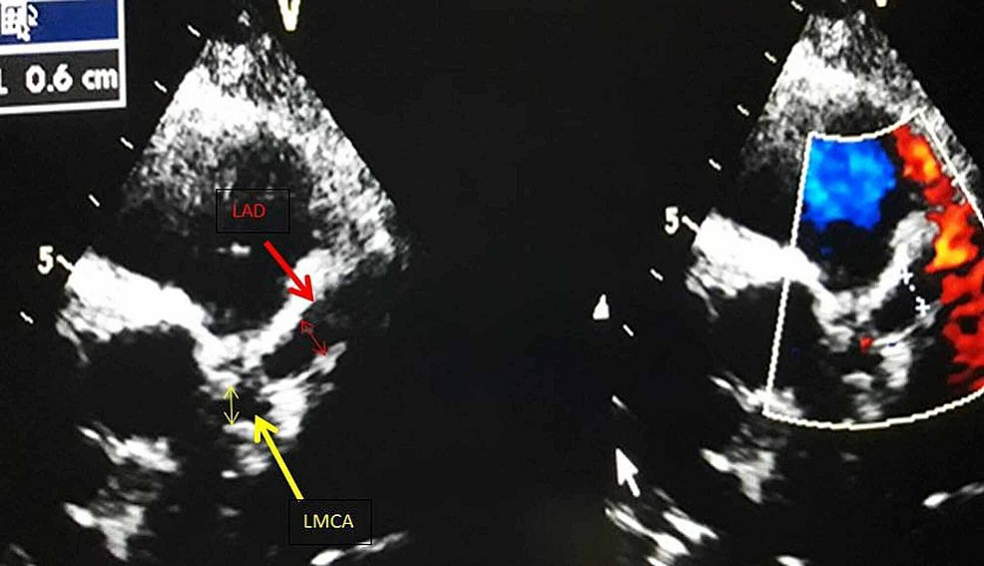
Echocardiography: parasternal short axis view showing left main coronary artery (LMCA) 6mm, left anterior descending artery (LAD) 6.2mm

**Figure 2:**
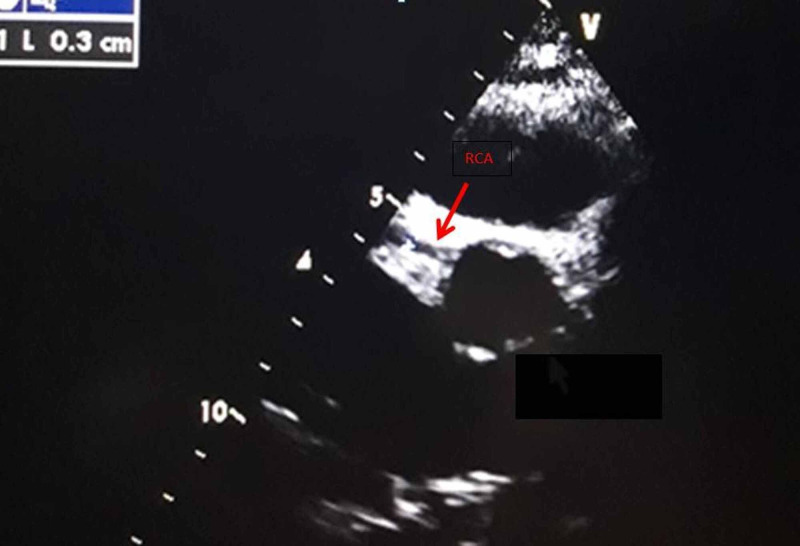
Echocardiography: parasternal short axis view showing right coronary artery (RCA) 3mm

In view of persisting fever, lack of response to antibiotics, cervical lymphadenopathy, oral changes, hand and feet edema with elevated inflammatory markers (↑CRP, ↑ESR), atypical Kawasaki disease was suspected and echocardiography was done. Echocardiography showed coronary artery dilatation with the left main coronary artery (LMCA) 6mm (Z score 6.4) [7], left anterior descending artery (LAD) aneurysm 6.2mm (Z score 9.04), right coronary artery (RCA) 3mm (Z score 0.53) with good biventricular function (ejection fraction [EF] 67%) (Figure 1, 2). American Heart Association (AHA) guidelines describe coronary artery aneurysms (CAA) as small (<5mm internal diameter), medium (5-8mm) and giant (>8mm) [4]; Z-scores in the LAD or RCA of 2.5 or greater are diagnostic of CAA [8].

The child was started on intravenous immunoglobulin (IVIG) at 2 gram/Kg body weight over 12 hours and tab aspirin was started at an anti-inflammatory dose at 80 mg/Kg in four divided doses. Gradually the child improved with subsidence of fever in 36 hours after IVIG therapy, decreased edema and lymph node size by the fourth day. Aspirin was decreased at anti-platelet dose of 3-5 mg/kg daily single dose after 72 hours of subsidence of fever. The child was discharged on day 13 of admission. Before discharge, the blood test was repeated which showed improvement with TLC 9700/cmm, platelet count 430000c/mm, and CRP 3.58. At the time of discharge desquamation of palms and soles was seen towards the end of the second week.

On follow-up in the outpatient department, the echocardiographic findings at two weeks were unchanged. At six weeks, regression in the coronary artery was seen with LMCA 4mm, LAD 5mm, RCA 2mm, and at three months LMCA 3mm, LAD 5mm, RCA 3 mm; tablet aspirin was continued and follow-up at six months showed complete improvement in coronary artery diameter with LMCA 2.5mm (Z score-1.21), LAD 3mm (Z score 1.51), RCA 2.8mm (Z score 0.12). Two years later the boy is in good condition without any cardiac sequela. 

## Discussion

The diagnosis of incomplete or atypical KD is challenging, especially in children who do not fulfill the clinical criteria. Some children with KD present with unusual features like pyuria, meningitis, or shock [9]. According to AHA recommendations, incomplete or atypical KD should be considered in patients with fever for more than five days and two or three compatible clinical criteria associated with cardiac findings on echocardiography and supplemental laboratory criteria (anemia, thrombocytosis >450,000 after the seventh day of fever, decreased albumin level <3 g/dL, elevated alanine aminotransferase, leukocytosis ≥15,000/mm³, and pyuria ≥10 WBC/hpf) [6]. In the case we describe here, a nine-year-old boy presented with fever, lymphadenopathy, parotitis, and torticollis. Initial diagnosis was infective parotitis in this case. Mumps and parotid abscess were considered.

The unusual presentations often result in a delay in the diagnosis. Parotitis is one such unusual presentation of Kawasaki disease. In a study, 15 cases of Kawasaki disease occurring with or just after parotitis have been reported [9]. Head and neck manifestations of KD are highly variable and may present with mastoiditis, cervical adenitis, upper airway obstruction, pharyngitis, acute tonsillitis, torticollis, retropharyngeal abscess-like, parapharyngeal abscess, and peritonsillar abscess [10].

Torticollis in this seemed to be due to painful cervical lymphadenitis. This is unusual for KD, although there are reports of KD presenting with torticollis [11]. The rate of cervical lymphadenopathy occurring as the initial presenting symptoms is only approximately 12% [12]. In our patient, cervical lymphadenopathy appeared as the first and most prominent symptom.

Our patient also developed gastrointestinal symptoms during his hospital stay. In a review study of gastrointestinal involvements among KD patients, Colomba et al. showed that the most common gastrointestinal manifestations were abdominal pain, vomiting, and diarrhea [13]. A study from Iran by Nasri et al. among 201 cases of KD with gastrointestinal manifestation of KD showed that a considerable percentage of KD patients had gastrointestinal problems such as abdominal pain (17.4%), diarrhea (16.9%), and vomiting (28.9%) [14].

We suspected KD in this child on the eighth day of fever and did an echocardiography that revealed a coronary artery aneurysm. Atypical age (less than one year or over 10 years) of presentation of KD is more commonly associated with aneurysm or dilatation of coronary arteries [15]. Despite treatment with IVIG in the dose of 2 gm/kg body weight, 5% of children can develop coronary artery aneurysm [13]. Giant aneurysm, based on Japanese Ministry of Health criteria [3], may be seen in 1% of cases. Coronary artery aneurysms show angiographic regression in nearly 50% of cases within one to two years following the illness, with smaller lesions having a greater chance of resolution [6].

Delayed diagnosis due to atypical presentation resulted in delay in treatment in our case, as our patient did not meet the classical criteria for the diagnosis of KD until the eighth day of illness. Untreated children develop coronary artery aneurysms rather commonly. Fortunately our child has remained well since discharge despite delayed treatment and is under follow-up at pediatric cardiology outpatient department (OPD).

## Conclusions

A high index of suspicion should be maintained in children presenting with fever and unusual manifestations like lymphadenopathy and parotitis, especially where empiric antibiotics are ineffective. Evaluation of cardiac function and coronary artery status with echocardiography is helpful in defining the diagnosis of KD in such cases. As it is a noninvasive test, it should be undertaken at the first possible clinical suspicion.

## References

[1] Jennette JC, Falk RJ, Bacon PA (2013). 2012 revised International Chapel Hill Consensus Conference nomenclature of vasculitides. Arthritis Rheum.

[2] Singh S, Kawasaki T (2009). Kawasaki disease - an Indian perspective. Indian Pediatr.

[3] McCrindle BW, Rowley AH, Newburger JW (2017). Diagnosis, treatment, and long-term management of Kawasaki disease: a scientific statement for health professionals from the American Heart Association. Circulation.

[4] Newburger JW, Takahashi M, Gerber MA (2004). Diagnosis, treatment, and long-term management of Kawasaki disease: a statement for health professionals from the Committee on Rheumatic Fever, Endocarditis and Kawasaki Disease, Council on Cardiovascular Disease in the Young, American Heart Association. Circulation.

[5] Momenah T, Sanatani S, Potts J, Sandor GG, Human DG, Patterson MW (1998). Kawasaki disease in the older child. Pediatrics.

[6] Taubert KA, Rowley AH, Shulman ST (1994). Seven-year national survey of Kawasaki disease and acute rheumatic fever. Pediatr Infect Dis J.

[7] Dallaire F, Dahdah N (2011). New equations and a critical appraisal of coronary artery Z scores in healthy children. J Am Soc Echocardiogr.

[8] Yu JJ (2012). Diagnosis of incomplete Kawasaki disease. Korean J Pediatr.

[9] Li Y, Yang Q, Yu X, Qiao H (2019). A case of Kawasaki disease presenting with parotitis: a case report and literature review. Medicine (Baltimore).

[10] Yoskovitch A, Tewfik TL, Duffy CM, Moroz B (2000). Head and neck manifestations of Kawasaki disease. Int J Pediatr Otorhinolaryngol.

[11] Dyer T, Dancey P, Martin J, Shah S (2018). Torticollis as presentation for atypical Kawasaki disease complicated by giant coronary artery aneurysms. Case Rep Pediatr.

[12] Hung MC, Wu KG, Hwang B, Lee PC, Meng CC (2007). Kawasaki disease resembling a retropharyngeal abscess -- case report and literature review. Int J Cardiol.

[13] Colomba C, La Placa S, Saporito L (2018). Intestinal involvement in Kawasaki disease. J Pediatr.

[14] Nasri P, Adibmajlesi Z, Rahimi H (2020). Gastrointestinal manifestations in children with Kawasaki disease in Isfahan, Iran. Arch Pediatr Infect Dis.

[15] de La Harpe M, di Bernardo S, Hofer M, Sekarski N (2019). Thirty years of Kawasaki disease: a single-center study at the University Hospital of Lausanne. Front Pediatr.

